# Attitudes and Behaviors of Practicing Community Pharmacists Towards Patient Counselling

**DOI:** 10.4103/0250-474X.56029

**Published:** 2009

**Authors:** R Adepu, B. G. Nagavi

**Affiliations:** Department of Pharmacy Practice, J. S. S. College of Pharmacy, S. S. Nagara, Mysore-570 015, India; 1 Dean, College of Pharmacy, RAK Medical and Health Sciences University, Ras Al-Khaima, UAE

**Keywords:** Attitudes and behaviors, community pharmacists, patient counseling, patient information leaflets

## Abstract

The present study was conducted to assess the attitudes and behaviors of practicing community pharmacists towards patient counselling and use of patient information leaflets in the state of Karnataka. Convenient sampling method was adopted to collect the responses with the help of self-completion questionnaires. A total of 258 practicing community pharmacists in the age group of 22–60 y of both gender with practicing experience of 2-30 y participated in the study. Majority of respondents (80%) agreed that, patient counselling is their professional obligation. About 17% of the respondents mentioned that, they try to give basic information regarding drug usage to the patient. The reasons stated by the pharmacists to provide patient counselling were, professional satisfaction (43%), patients go with satisfaction (32%), observed increase in sales (8%), and also improved patient compliance (7.5%). The major barriers for offering patient counselling were mentioned as pharmacists' inadequate knowledge and confidence (78%), doctor dispensing (72%), no professional fee (56%), poor response from patients (82%), inadequate continuous professional development programs (75%). Many respondents agreed that, patient information leaflets certainly help in counselling but available information leaflets are company generated and prescriber focused. Many respondents found the present continuing professional development module was useful and are interested in weekend workshops to update their professional knowledge (83%). Restrictions on doctor dispensing, legalization of patient counselling, regular continuing professional development programs are the factors observed to motivate the pharmacists to offer patient counselling.

Pharmaceutical care concept is well received by the pharmacists around the world[1]. Pharmacists in developed countries working in hospitals and community are providing pharmaceutical care services to their patients to improve the quality of life[2]. Patient counselling is considered as an important component of pharmaceutical care services[3]. In most of the developed countries, patient counselling is regarded as an important professional responsibility of the pharmacists and in some countries it is mandatory[4]. United States Pharmacopoeia has identified and listed barriers for patient counseling in community pharmacies[5]. One of the important barriers of counselling are provider based, i.e. the pharmacist. Lack of knowledge, lack of time, lack of training, lack of interest, lack of remuneration are the important reasons expressed by pharmacists in some international studies[6]. In India, there are about 0.6 million pharmacists serving in the community. Most of them are with basic qualification of Diploma in Pharmacy (D. Pharm.), which is a two-year course after 12 y of schooling. Majority of practicing pharmacists are confined to trade activities in the community and engage only in filling the prescriptions. Some pharmacists may provide medication usage information to the patients only on request.

As per section 42 of 1948 Pharmacy Act, only qualified persons are permitted to sell the pharmaceutical formulations[7]. In order to induce the professional competency, Pharmacy Council of India (PCI) introduced Educational Regulations (ER) in 1953 for standardizing Diploma in Pharmacy education[8]. Many changes were brought in the curriculum of Diploma in Pharmacy over the last five decades, to improve the knowledge base in budding pharmacists. Many professional subjects such as community pharmacy and health education, community pharmacy management, and hospital and clinical pharmacy were introduced at the Diploma in Pharmacy level in the latest ER-91. Bachelor of Pharmacy (B. Pharm.) curriculum in many Indian Universities is predominantly industry focused. In recent years, suitable and need based amendments were made in Bachelor of Pharmacy curriculum that resulted in the introduction of pharmacy practice subjects with an aim to motivate new graduates to take up jobs in hospitals and community set up.

Despite efforts by statutory bodies, registration and regulatory authorities, pharmacists in community pharmacies are still confined themselves to trade than blending the trade with profession. An occupation having certain special attributes such as knowledge and autonomy and provide services to the clients is called as “Profession”[9].

Many Indian community pharmacists consider pharmacy is only trade rather the profession. In a study conducted regarding public perception about pharmacists, 92% of the respondents rated community pharmacists as drug traders[10]. In another study regarding the pharmacists' awareness about professional responsibilities, more than 50% of the respondents are unaware about their professional responsibilities[11].

India is a country with significant drug related problems due to poly pharmacy such as drug duplication, under dose, over dose, potential drug interactions[12]. Illiteracy, inadequate information about medications usages are the major reasons[13]. Due to heavy patients load, doctor's consultancy limits to the issue of prescriptions to patients with very limited or no information about medications and their use. Pharmacists who are supposed to be the information providers and should act as vital link between patients and the prescribers are remaining as the prescription fillers. This lenient attitude of the pharmacists motivated us to probe the attitudes and behaviors of pharmacists towards patient counseling.

## MATERIALS AND METHODS

The present study was conducted in the state of Karnataka. To assess the attitudes and behaviors of practicing community pharmacists, a suitably designed questionnaire was developed comprising 48 questions on personal details, pharmacy details, awareness about professional responsibilities, and attitude towards patient counseling and use of information leaflets.

Karnataka is one of the south Indian states with population over 60 million spread in 29 districts[14]. About 41,345 pharmacists are registered to the Karnataka State Pharmacy Council (KSPC) and working in hospital and community pharmacies[15]. The present study was conducted using convenience-sampling method. In nine district head quarters, a weekend workshop on pharmaceutical care and patient counselling was organized with the support of District Chemists and Druggists Associations. Before beginning the seminar, the questionnaires were distributed to the enrolled members with a request to fill all contents of the questionnaire. After the work shop, a feedback regarding the content of the program, and its usefulness was collected. The collected data was analyzed.

## RESULTS AND DISCUSSION

Two hundred and fifty eight practicing community pharmacists participated in the study from nine district head quarters of Karnataka state covering east, west, south, north and central parts of Karnataka. Among the participants 93% were males in the age group of 26-60 y with practice experience of 2-30 y and 7% were females in the age group of 25-45 y with practice experience of 5 to 15 y. Majority of the participants were with ER-91 D. Pharm. qualification (56.3%), and Sole proprietor pharmacists were 55.03%. From the details provided by the pharmacists, it was observed that 4% and 9% pharmacies did not meet the minimum space requirement and storage conditions for biological preparations. About 94% of pharmacies did not possess computers, and 81% pharmacies did not have an identified place for patient counselling (Table 1).

**TABLE 1:** DEMOGRAPHIC DETAILS OF THE RESPONDENTS (N=258)

Demographic item	Percentage
Crash course	3.0
D. Pharm (E.R.-81)	27.0
D. Pharm (E.R.-91)	56.0
B. Pharm	4.0
M. Pharm	1.0
Others	9.0
Respondent's Position	
Sole Proprietor pharmacist	55.0
Partner pharmacist	13.0
Pharmacy In charge	7.0
Permanent Full time Pharmacist	19.0
Permanent part time Pharmacist	4.0
Reliever/casual	2.0

Many participants have mentioned that, they are aware of some professional services such as patient counselling, responding to symptoms of common ailments and health promotion (Table 2) but not practicing the same because of various reasons such as lack of time, knowledge and confidence, no professional fees, doctors dispensing and poor response from patients (Table 3). About 17% of the respondents mentioned that they try to give some basic information about the product, its usage technique and so on mainly because of personal interest and observed that patients go with satisfaction (Table 4). Only 15% of the pharmacists mentioned that patient information leaflets (PIL) are useful during the counselling. Regarding the content of the workshop and its usefulness, about 90% of the participants mentioned that the workshop was very much informative and useful and they also mentioned that, at least once in three months, such workshops should be organized (Table 5). The respondents felt that the workshops gave them an opportunity to update their knowledge on diseases and drugs. They also mentioned that, the role plays were very useful in improving their communication skills and learned the technique of counselling.

**TABLE 2:** AWARENESS ABOUT THE PROFESSIONAL RESPONSIBILITY

Professional Services	Percentage
Processing of the prescriptions	27.0
Maintaining patient medication records	11.0
Patient counselling	61.0
Drug information services	48.0
Responding to symptoms of minor ailments	72.0
Health promotion on communicable diseases	67.0
Family planning promotion	33.0
Immunization programs	29.0

**TABLE 3:** REASONS FOR NOT COUNSELLING

Reasons	Percentage
Lack of Time	84
Lack of Knowledge & Confidence	78
Lack professional fee	56
Poor response from patients	82
Doctor dispensing	72
No Continuous Professional Development programs	75

**TABLE 4:** REASONS FOR OFFERING PATIENT COUNSELING

Reasons	Percentage
Gives Professional satisfaction	53.0
Patients go with satisfaction	32.0
Observed increased sales	8.0
Observed improved patient compliance	7.0

**TABLE 5:** PREFERRED CHOICE OF CONTINUOUS PROFESSIONAL DEVELOPMENT (CPD) PROGRAMS

Preferred Choice of CPD	Percentage (N = 258)
Correspondence course	16.0
Monthly seminars	50.0
Regular class room type of teaching	1.0
Weekend workshops	30.0
Evening tutorials	1.0
Online training	2.0

Pharmacy practice is in a rudimentary stage in most of the developing countries. In India, recently practice concept has gained momentum with the success of clinical pharmacy program at J. S. S. Hospital, a south Indian teaching hospital in Mysore. Considerable amount of efforts were made by the Pharmacy Council of India (PCI), a professional statutory body from the past five decades in developing the pharmacy practice in India through restructuring the curriculum and up grading the minimum registrable qualification. Due to various reasons only restructuring of the curricula was possible but up gradation of minimum registrable qualification has not taken place yet.

Although PCI has introduced important professional subjects at diploma and undergraduate levels, still professional services offered by pharmacists are negligible. In community pharmacies, trader attitude is more predominant in the pharmacists than professional behaviour. Reason for this is, majority pharmacists in the community, possess a two year diploma in pharmacy qualification. The knowledge base of these persons is very limited especially people from crash course and ER-81 scheme. The same is reflected in the present study. Many pharmacists were not clear about their professional responsibilities and did not meet the legal requirements such as space and storage conditions for biological products. In one of our studies, we found that about 35% of pharmacies are owned and run by non-pharmacists who hire the licenses of Qualified Persons to meet section 42 of Pharmacy Act 1948 and behave as trader[7]. As per section 42 of Pharmacy Act 1948, certificate of qualified person is compulsory to open a pharmacy. Lenient behaviour of drug enforcement officers may be one of the reasons for this situation.

As per the latest census, 35% of populations are still illiterates[16]. Many times, due to lack of awareness about the medication importance and its usage, incidents were there, where some village women swallowed peassaries and antibiotics were taken just for two to three days. Such drug related and disease management problems can be overcome by effective patient counselling. Worldwide many studies have also proven this[17,18].

In the present study, though many pharmacists mentioned that, patient counselling is their professional responsibility but due to various reasons only few pharmacists are actually providing information to the patients about medication usage. Analysis of poor patient counselling attitudes of the pharmacists suggests that, pharmacists are not entitled for any additional professional fee for dispensing medications and offering professional services such as patient counselling.

In 1970, Kelkar committee report recommended a small fee of half a rupee for dispensing one prescription[19] but due to unknown reasons the report was not implemented. In the feedback session many pharmacists expressed that, since patient counselling is not legalized, they cannot charge any extra amount for the information provided to the patient and also for dispensing. If any extra amount is charged and collected by oversight through bill, if the matter is brought to the notice of local drugs Inspector, pharmacist may be penalized heavy amounts of penalties and sometimes even cancellation of their license. In addition to this, many pharmacists expressed that, during peak business hours, it is very difficult to counsel patients because of patients' rush. Another reasons observed are, lack of knowledge and confidence, inadequate continuous professional development programs, doctor dispensing.

State pharmacy councils under the direction of PCI registers pharmacists and grants licenses enabling them to practice. Unlike pharmaceutical societies in developed countries, state pharmacy councils in India do not have prescribed professional standards for practicing pharmacists, and guidelines to practice the profession. Recently some state pharmacy councils have started drug information centers and started conducting continuing education programs for pharmacists. But the frequency of seminars is very limited, may be once in a year and majority participants are mainly from cities. Increased number of Continuous Professional Developments (CPDs) and motivating all the pharmacists in the state to attend minimum number of CPDs may solve the problem of lack of knowledge and confidence. In the present study, pre and post test revealed the changes in the entry and exit level behaviours of the participants. In conducting CPDs in district headquarters, state pharmacy councils may utilize the subject expertise from local pharmacy colleges. This kind of arrangement in the long run may benefit both state pharmacy councils and the teaching institutions in terms of quality education, prescribing and implementing professional standards, and sharing the monetary benefits.

Another hurdle found in patient counselling is Doctor dispensing. Schedule K of 1940 Drugs and Cosmetics Act[20] permitted doctors in the early days of independence to dispense medication to their patients in the rural areas where pharmacists were not available. Now the situation is changed. We have adequate number of pharmacies in the community to meet the drug requirements of over one billion population. Since the last two decades, the problem of doctor dispensing is rising due to commercial interest especially in cities and becoming a big threat to the survival of many pharmacists. This problem needs to be addressed with policy makers to put an end to the doctor dispensing as the pharmacist and patients' ratio is satisfactory in the society at the moment.

In many studies, Patient information leaflets are found to be useful in strengthening the counseling process[21]. In India, pharmacist generated information leaflets are available in very limited number. Some pharmacists provide company generated information leaflets to their patients, which are doctor oriented. Research on development of information leaflets incorporating suitable readability, lay out, and design is the immediate need that will help pharmacists to offer useful information leaflets to their patients during the counselling.

A Six-hour training module developed for conducting workshop. This consists of topics on pharmaceutical care, patient counseling, pathophysiology, and therapeutics of one chronic disease such as hypertension, Asthma and diabetes mellitus and a role-play on patient counselling. This workshop was more like an interaction session, where every participant was encouraged to participate in the discussion and role-play. Thus many participants found the workshop was very useful. Post training test scores prove this.

From the study it can be concluded that, 20% participating pharmacists were unclear about their professional responsibilities. Though 61% of participating pharmacists said that, patient counselling is their professional responsibility, but only few pharmacists were offering this service. Major hurdles listed by respondents in offering patient counselling services are, lack of knowledge and confidence in practicing pharmacists, no professional fee, and doctor dispensing. Reasons for some pharmacists offering patient counselling are mainly because of professional satisfaction. Majority participants found the training module is informative and useful.
